# Negative Regulation of Pulmonary Th17 Responses by C3a Anaphylatoxin during Allergic Inflammation in Mice

**DOI:** 10.1371/journal.pone.0052666

**Published:** 2012-12-20

**Authors:** Hoyong Lim, Young Uk Kim, Scott M. Drouin, Stacey Mueller-Ortiz, Kyoungah Yun, Eva Morschl, Rick A. Wetsel, Yeonseok Chung

**Affiliations:** 1 Center for Immunology and Autoimmune Diseases, Institute of Molecular Medicine, the University of Texas Medical School at Houston, Houston, Texas, United States of America; 2 Graduate School of Biomedical Sciences, the University of Texas Health Science Center, Houston, Houston, Texas, United States of America; 3 Daejon Health Sciences College, Daejon, South Korea; New York University, United States of America

## Abstract

Activation of complement is one of the earliest immune responses to exogenous threats, resulting in various cleavage products including anaphylatoxin C3a. In addition to its contribution to host defense, C3a has been shown to mediate Th2 responses in animal models of asthma. However, the role of C3a on pulmonary Th17 responses during allergic inflammation remains unclear. Here, we show that mice deficient in C3a receptor (C3aR) exhibited (i) higher percentages of endogenous IL-17-producing CD4^+^ T cells in the lungs, (ii) higher amounts of IL-17 in the bronchoalveolar lavage fluid, and (iii) more neutrophils in the lungs than wild-type mice when challenged with intranasal allergens. Moreover, adoptive transfer experiments showed that the frequencies of antigen-specific IL-17-producing CD4^+^ T cells were significantly higher in the lungs and bronchial lymph nodes of C3aR-deficient recipients than those of wild-types recipients. Bone-marrow reconstitution study indicated that C3aR-deficiency on hematopoietic cells was required for the increased Th17 responses. Furthermore, C3aR-deficient mice exhibited increased percentages of Foxp3^+^ regulatory T cells; however, depletion of these cells minimally affected the induction of antigen-specific Th17 cell population in the lungs. Neutralization of IL-17 significantly reduced the number of neutrophils in bronchoalveolar lavage fluid of C3aR-deficient mice. Our findings demonstrate that C3a signals negatively regulate antigen-specific Th17 responses during allergic lung inflammation and the size of Foxp3^+^ regulatory T cell population in the periphery.

## Introduction

Allergic airway inflammation is a chronic life-threatening lung disease. The ‘chronic’ property of lung inflammation is tightly associated with allergen-specific adaptive immunity, especially CD4^+^ helper T cell responses. The contribution of Th2 cells during the allergic response to airway allergens via the production of IL-4, IL-5, IL-9 and IL-13 is now well established [1], [2], [3]. In addition to Th2 cells, recent advances have revealed a critical and non-redundant role of IL-17-producing CD4^+^ T cells (Th17) in lung inflammation [4], [5], [6].

Th17 cells have been characterized as a distinct lineage of helper T cells that are programmed by transcription factors RORγt and RORα [7], [8]. While TGF-β and IL-6 are essential for initial Th17 lineage commitment, IL-23 and IL-1 are required for functional maturation of Th17 cells *in vivo* [9], [10], [11], [12]. Differentiated Th17 cells mediate diverse pro- and anti-inflammatory functions *in vivo* through the production of signature cytokines including IL-17 (IL-17A), IL-17F, IL-22, and IL-26 [13]. For instance, Th17 immunity has been described to be protective against various bacterial and fungal infections [10]. On the other hand, accumulating evidence demonstrated critical pathogenic role of Th17 responses in chronic inflammatory disorders, such as rheumatoid arthritis, psoriasis, and multiple sclerosis in experimental animal models as well as in humans [14], [15], [16], [17], [18], [19]. Of importance, treatment with anti-IL-17 antibodies has been shown to ameliorate clinical symptoms of psoriasis, and arthritis in clinical trials [20], [21], [22]. Therefore, targeting Th17 cytokines may provide a promising therapeutic approach for the treatment of numerous chronic inflammatory human diseases.

Increased levels of IL-17 were detected in the lung, sputum and bronchoalveolar lavage (BAL) fluids of asthmatic patients [23], [24], [25], suggesting a possible involvement of Th17 cells in asthma. While Th2 responses promote eosinophilic inflammation in the lungs [26], [27], Th17 responses have been suggested to play a non-redundant role in pulmonary inflammation by inducing neutrophilic inflammation. Elevated neutrophilia is correlated to asthma severity [28], [29], [30]. Supporting this notion, recent studies have shown that the IL-17 from pulmonary T cells enhances airway hyper-responsiveness (AHR) and neutrophilic inflammation in animal models of asthma [31], [32], [33], [34], [35]. On the other hand, it has been shown that neutralizing IL-17 augments allergic responses in the lung, and that administration of IL-17 ameliorates eosinophilia and airway hypersensitivity in an animal model of asthma [36], suggesting that IL-17 suppresses lung inflammation. In addition, the negative regulation of allergic lung inflammation by IL-17-producing γδ T cell has been described [37]. Thus, the biological roles of Th17 responses in allergic lung diseases are presently not well defined, and the overall impact of Th17 cells in allergic asthma remains controversial. The cellular and molecular mechanisms mediated by Th17 cells during allergic asthma are likely complex; therefore, extensive further investigation will be required before the overall picture of how Th17 cells influence the allergic response to lung allergens can be fully visualized.

The complement system is primarily known for its crucial host defense against bacterial and viral infections through opsonization and formation of the membrane attack complex [38], [39], [40]. Activation of complement by invading pathogens generates various cleavage products including the anaphylatoxins C5a and C3a [41], [42]. C3a mediates diverse functions in the immune system upon binding to its receptor C3aR, which is expressed on certain parenchymal cells, such as lung epithelial cells, and on numerous myeloid cells including neutrophils, macrophages, mast cells and basophils [43], [44], [45], [46], [47], [48]. Patients with asthma exhibit elevated levels of C3a in the sera as well as in the airway [49], [50], [51]. C3aR-deficient (C3aR^−/−^) mice exhibit a decreased number of eosinophils in the airway with reduced Th2 responses [52] and less airway hyperresponsiveness [50] in experimental asthma models. In addition, administration of C3aR antagonist ameliorates the airway inflammation induced by allergens in mice [53], [54]. Although these previous studies have made a strong case for C3a as a pathogenic mediator of allergic lung disease, only recently has the impact of C3a on IL-17 in the context of allergic asthma been investigated. For instance, it has been recently shown that C3aR^−/−^ mice produce less IL-17 when challenged with house dust mite allergens than wild-type challenged mice [5]. In the same study, it was demonstrated that C3a promotes IL-17 production upon allergenic challenge in the lung by suppressing IL-10 production while inducing IL-23 from dendritic cells. However, in the inflamed lung, IL-17 can be generated by CD4^+^ T cells as well as innate immune cells including γδ T cells, NKT cells, and alveolar macrophages [55], [56], [57]. Since the C3aR has been reported to be expressed on macrophages and other bone marrow derived cells, the C3a-dependent IL-17 phenotype observed in this dust mite mouse model may be due to more extensive cellular/C3a interactions than just dendritic cells.

Accordingly, we sought in the current study to define more comprehensively the role of C3a signaling on the generation of allergen-specific Th17 cells by employing C3aR^−/−^ mice in a well-established animal model of allergic lung inflammation [58], [59], [60], [61]. In contrast to a previous report [5], we observed a significantly higher frequency of allergen-specific Th17 cells in the lungs and lymph nodes of C3aR^−/−^ mice than those of wild-type mice. The increased pulmonary Th17 responses in C3aR^−/−^ mice was independent of the increased regulatory T cell population but dependent on C3aR signaling on hematopoietic cells.

## Materials and Methods

### Mice

C57BL/6, B6.SJL and OT-II mice were purchased from Jackson Laboratories (Bar Harbor, ME). CD45.1^+^OT-II mice were generated by crossing B6.SJL and OT-II mice. C3aR^−/−^ mice were generated as described previously [62] and have been fully backcrossed onto the C57BL/6 background. All mice were maintained in the specific pathogen free facility at the vivarium of the Institute of Molecular Medicine. All animal experiments were performed using protocols approved by Institutional Animal Care and Use Committee of the University of Texas at Houston.

### Animal models of allergen-induced lung inflammation

Allergic lung inflammation was induced by repeated intranasal challenges with model allergens by adopting an established model of allergic asthma [58], [59], [60], [61]. In brief, mice were anesthetized with isoflurane and were intranasally administered with a mixture of 7 μg of *Aspergillus melleus* proteinase (Sigma, St Louis, MO) and 20 μg of OVA (Grade V, Sigma) in a volume of 50 μl every other days (day 0, 2, 4, 6) four times total. In some experiments, we administered heat-inactivated *Aspergillus* proteinase prepared by boiling the proteinase for 10 min. In some experiments, CD4^+^ T cells purified from CD45.1^+^OT-II mice were intravenously transferred on day −1 (4–5×10^6^ cells/mouse) to track antigen-specific T cell responses. Twenty-four hours after the last treatment, all mice were euthanized with CO_2_, and BAL fluid and lung and bronchial lymph nodes were obtained for analysis. In regulatory T cell depletion studies, we intraperitoneally injected mice with 150 μg of anti-CD25 mAb (clone PC-61, BioXcell, West Lebanon, NH) or control IgG for three times (day −2, 0, 2) [63]. In some experiments, we intraperitoneally injected mice with 100 μg of anti-IL-17A mAb (clone TC11-18H10.1, BioLegend, San Diego, CA) or control IgG for three times (day 0, 2, 4) into C3aR^−/−^ mice to examine the role of IL-17 *in vivo*.

### Bone marrow reconstitution study

Bone marrow reconstituted mice were generated as described previously [64]. In brief, bone marrow cells were obtained from C3aR^−/−^ or wild-type mice, and were intravenously transferred (1×10^7^ cells/mouse) into lethally irradiated C3aR^−/−^ or wild-type mice (950 rad). Six to eight weeks later, the reconstituted mice were intravenously injected with purified CD45.1^+^OT-II T cells, followed by intranasal challenges with allergen as described above.

### Analysis of bronchoalveolar lavage (BAL) fluid

After the trachea was cannulated, the air lumen was washed twice with 0.7 ml of PBS containing proteinase inhibitor cocktail (GenDepot, Houston, TX), as described previously [65]. Collected BAL fluid was centrifuged at 100×g for 3 min at 4°C. The supernatant was collected and IL-17 concentration determined by ELISA. BAL cells in the pellet were resuspended with PBS, centrifuged onto microscope slides by cytospin, and were stained with Diff-Quik (Siemens, Newark, DE). Differential cell counts were performed to measure cellular infiltration into the airway by counting at least 300 cells per slide, and absolute numbers of each cell population were calculated based on total cell counts.

### Isolation of cells from the lungs

To obtain lymphoid cells in the lungs, we surgically removed the lungs after withdrawal of blood by heart puncture, and teased them into small pieces by scissors and incubated them in RPMI 1640 medium containing 10% of FBS (Invitrogen, Grand Island, NY), 0.5 mg/ml of collagenase (Roche, Switzerland), 2 mg/ml of Dispase (Invitrogen), and 30 μg/ml of DNase I (Sigma-Aldrich) for 40 min at 37°C in a CO_2_ incubator. The cells were agitated every 10 min by pipetting. The cells were washed with PBS, filtered through 120 μm nylon mesh, resuspended with PBS containing 1.5 % FBS. Lymphoid cells were separated by density separation by using lymphocytes separation medium (MP Biomedicals, Solon, OH). Cells in the interphase were harvested and washed twice with 1.5% FBS in PBS.

### Flow cytometry

Cells isolated from mice were incubated for 3–4 hours with PMA (100 ng/ml, Sigma-Aldrich) and ionomycin (1 μM, Sigma-Aldrich) in the presence of Brefeldin A and Monensin (all from eBioscience, San Diego, CA) [12]. Cells were washed with PBS containing 1.5% FBS, and incubated with anti-CD16/CD32 antibody (2.4G2). After washing, the cells were stained with PerCp-Cy5.5-conjugated anti-CD4 mAb (clone GK1.5, BioLegend, San Diego, CA) and Pacific Blue-conjugated anti-CD45.1 mAb (clone A20, BioLegend) for surface staining. Cells were washed and resuspended in permeabilization buffer (eBioscience) for 30 min at 4°C, followed by staining with PE-conjugated anti-IL-17A mAb (clone TC11-18H10.1, BioLegend), Alexa488-conjugated anti-IFN-γ mAb (clone XMG1.2, eBioscience), Alexa647-conjugated anti-IL-4 mAb (clone 11B11, BioLegend) and APC-conjugated anti-IL-5 mAb (clone TRFK3, BioLegend).

For detection of CD4^+^Foxp3^+^ regulatory T cells, cells were surface stained with PerCp-Cy5.5-conjugated anti-CD4 mAb, and incubated in Foxp3 staining buffer (eBioscience) for 30 min. Cells were washed and intracellular stained with Alexa488-conjugated anti-Foxp3 mAb (clone FJK-16 S, eBioscience). The stained cells were analyzed by FACSAria II or FACSCalibur flow cytometer (all from BD Bioscience, San Jose, CA). Data were processed using FlowJo software (TreeStar, Ashland, OR).

### Lung histology

The lungs were dissected after inflated with 0.5 ml of 10% buffered formalin (Sigma-Aldrich) and fixed overnight in formalin. After embedding in paraffin, lung sections (5 µm) were stained with hematoxylin and eosin (H&E) or with periodic acid Schiff (PAS) reagent, and lung inflammatory infiltrates and mucus producing cells were visualized by light microscopy (×20) (Carl Zeiss, Thornwood, NY) equipped with a SPOT-RT digital camera (Diagnostic instruments, Sterling Heights, MI).

### AHR measurements

Airway responsiveness to acetylcholine challenge was determined as previously described [52]. Briefly, 24 hours after the last challenge, mice were anesthesized and the tracheas were surgically exposed, cannulated with a blunt-ended, 20-gauge angiocatheter, and were ventilated with 100% oxygen at a rate of 150 breaths per min and a tidal volume of 9 μl/g before being placed into a rodent plethysmograph. Following paralysis with pancuronium bromide (4 μg/g), increasing doses of acetylcholine (0.1, 0.316, 1, 3.16 and 10 µg of solution per gram of body weight) was consecutively injected to the tail vein, and airway responses were measured by plethysmography (Buxco Electronics, Sharon, CT) for 1 min after injection of the each dose of acetylcholine.

### ELISA

BAL fluid and lungs from allergen challenged C3aR^−/−^ and wild-type mice as described above. Lung homogenates were obtained by using a homogenizer in the presence of proteinase inhibitor, followed by centrifuging at 120×g for 5 min at 4°C. Levels of cytokines and C3a in the BAL fluid and lung homogenates were determined by ELISA. ELISA kits for TGF-β (R&D systems, Minneapolis, MN), IL-17, IL-6 (BioLegend), and IL-23 (eBioscience) were used according to the manufacturer's instructions. Anti-C3a antibodies (clone 187–1162 and 187–419, BD Pharmingen) were used to measure C3a, as described previously [66].

### Quantitative real-time RT-PCR

Total RNA was extracted from total lung or spleen with TRIzol (Invitrogen) and reverse transcribed using amfiRivert reverse transcriptase (GenDepot) according to the manufacturer's protocol. Gene expression was measured with iTaq-SYBR Green Supermix (Bio-Rad Laboratories, Hercules, CA) and the ABI-PRISM 7900 detection system (Applied Biosystems, Foster City, CA). Data were normalized to expression of the β-actin gene. The following primer pairs were used: *C3* forward, 5′-CCAGCTCCCCATTAGCTCTG-3′; *C3* reverse, 5′-GCACTTGCCTCTTTAGGAAGTC-3′; *C3ar* forward, 5′- CCAGACACATCCACAGATGG-3′; *C3ar* reverse, 5′- TGTTTGCCAGTGTCTTCCTG-3′; *Il23a* forward, 5′-AAGTTCTCTC- CTCTTCCCTGTCGC-3′; *Il23a* reverse, 5′-TCTTGTGGAGCAGCAGATGTGAG-3′; *Il12a* forward, 5′-CCACCCTTGCCCTCCTAAAC-3′; *Il-12a* reverse, 5′-GGCAGCTCCC-TCTTGTTGTG-3′; *Il-12b* forward, 5′-CTTGCAGATGAAGCCTTTGAAGA-3′; *Il-12b* reverse, 5′-GGAACGCACCTTTCTGGTTACA-3′; *Il13* forward, 5′-GCTTATTGAGGAGCTGAGC-AACA-3′; *Il13* reverse, 5′-GGCCAGGTCCACACTCCATA-3′; *Il1b* forward, 5′-AAGGA-GAACCAAGCAACGACAAAA-3′; *Il1b* reverse, 5′-TGGGGAACTCTGCAGACTCAAACT-3′; *Il21* forward, 5′-TCATCAT-TGACCTCGTGGCCC-3′; *Il21* reverse, 5′-ATCGTACT-TCTCCACTTGCAATCCC-3′; *Csf2* forward, 5′-ATGCCTGTCACGTTGAATGAAG-3′; *Csf2* reverse, 5′-GCGGG-TCTGCACACATGTTA-3′; *Tnf* forward, 5′-GACGTGGAAGTGGC-AGAAGAG-3′; *Tnf* reverse, 5′-TGCCACAAGCAGGAATGAGA-3′; *Il6* forward, 5′-TATGAAGTTCCTCTCT-GCAAGAGA-3′; *Il6* reverse, 5′-TAGGGAAGGCCGTGGTT-3′; *Il10* forward, 5′-ATAACTGCACCCACTTCCCAGTC-3′; *Il10* reverse, 5′-CCCAAGTAA-CCCTTAAAGTCCTGC-3′; *Cxcl1* forward, 5′- TGGCTGGGATTCACCTCAAGAACA-3′; *Cxcl1* reverse, 5′- TGTGGCTATGACTTCGGTTTGGGT-3′; *Ccl7* forward, 5′- CTC ATA GCC GCT GCT TTC AGC ATC-3′; *Ccl7* reverse, 5′- GTC TAA GTA TGC TAT AGC CTC CTC-3′; *ACTB* forward, 5′-TGGAATCCTGTGGCATCCATGAAAC-3′; *ACTB* reverse, 5′-TAAAACGCAGCTCAGT-AACAGTCCG-3′.

### Statistical analysis

Data were analyzed with GraphPad Prism 5 (GraphPad Software, La Jolla, CA). Statistics were calculated with the two-tailed Student's t-test. *p* values of less than 0.05 were considered statistically significant.

## Results

### Expression of C3a and C3a receptor upon allergen challenge

**Figure 1:**
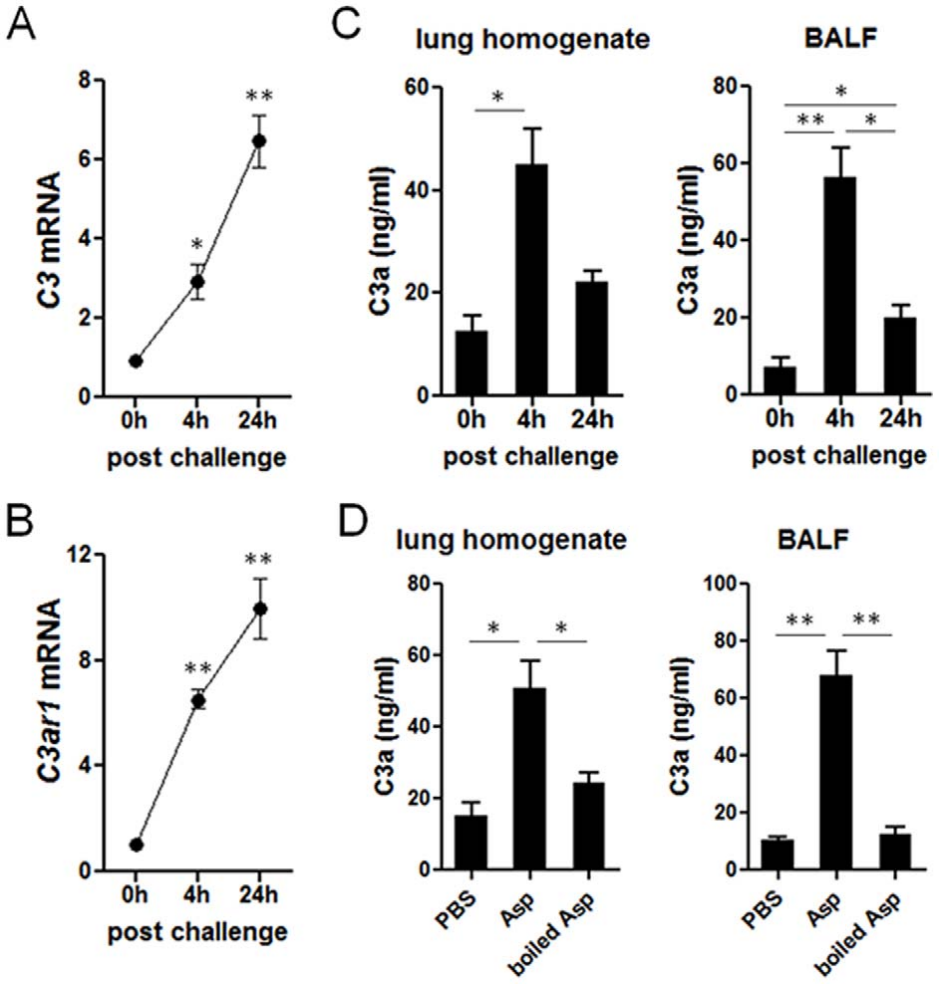
Expression of C3, C3a receptor and C3a in the lung upon allergenic challenge. C57BL/6 mice were intranasally challenged with the mixture of *Aspergillus* proteinase and OVA (*A-C*). The lungs and BAL fluid were collected from the treated mice (n = 3) at the indicated time points. C57BL/6 mice (n = 3) were intranasally injected with intact or boiled *Aspergillus* proteinase, and the lungs and BAL fluid were collected 24 hours after the treatment (*D*). The mRNA transcript levels of *C3* (*A*) and *C3ar1* (*B*) in the lungs were measured by quantitative RT-PCR. The concentration of C3a in the lung homogenate and BAL fluid was measured by ELISA (*C & D*). *, p<0.05 or **, p<0.01 in comparison with 0 hr time point.

Intranasal challenge with fungal-associated allergic proteinases has been well established to induce asthma-like allergic lung inflammation in mice [58], [59], [60], [61]. Therefore, we used the mixture of *Aspergillus* proteinase and chicken egg albumin (Asp/OVA) as our model allergen, as to address the role of C3a on pulmonary helper T cell responses. We first determined in naïve C57BL/6 mice changes in expression of C3, C3a, and its receptor C3aR following intranasal challenge with the model allergen. We observed significant increased expression of both *C3* and *C3ar1* transcripts in the lungs of allergen challenged mice compared to control mice as early as 4 hours after the challenge with further increases observed at 24 hours (Figure 1A & B). C3a protein was analyzed by ELISA. We observed a significant induction of C3a in the lung homogenates and BAL fluid of the challenged mice compared to the control mice, which peaked at 4 hours after the allergen challenge (Figure 1C). To test if the proteinase activity of the used allergen is required for the induction of C3a, we compared the production of C3a induced by intranasal injection of either intact or boiled *Aspergillus* proteinase. As depicted in Figure 1D, the amounts of C3a in the lung as well as in the BAL fluid were significantly reduced when mice were challenged with boiled allergen. Therefore, the proteinase activity of the model allergen is likely critical for the induction of C3a in the lung in this experimental setting.

### C3aR^−/−^ mice exhibited increased Th17 responses in the lung upon allergenic challenge

**Figure 2:**
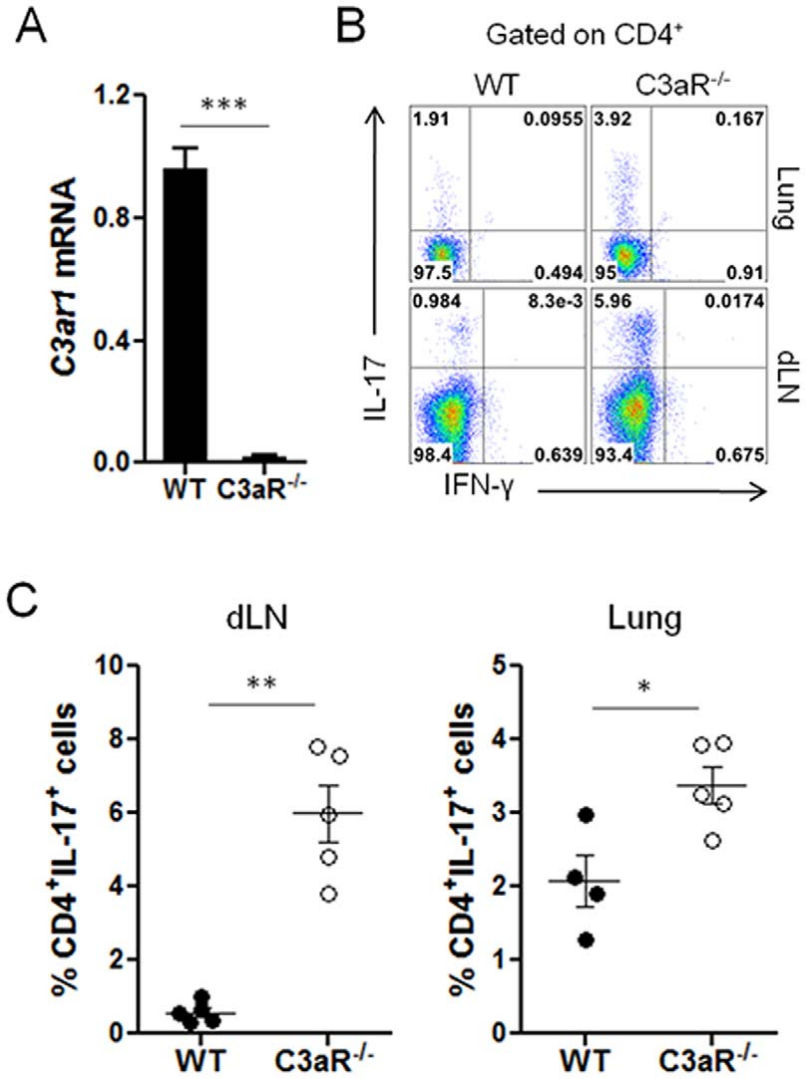
Increased frequency of IL-17-producing CD4^+^ T cells in C3aR-deficient mice upon intranasal challenges with allergen. *A*, The levels of *C3ar1* transcript in the splenocytes of wild-type and C3aR^−/−^ mice (n = 3) were analyzed by quantitative RT-PCR analysis. *B* and *C*, Groups of C3aR^−/−^ and wild-type mice (n = 4–5 per group) were intranasally administered with Asp/OVA every other days for four times. One day after the last challenge, lymphoid cells from the lung or draining LNs were obtained, restimulated with PMA and ionomycin in the presence of brefeldin A and monensin, and the expression of IL-17 and IFN-γ by CD4^+^ T cells was analyzed by intracellular staining. Data shown are gated on CD4 expression (*B & C*). Data shown are mean ± SE, and represent three independent experiments. *, p<0.05 or **, p<0.01 or ***, p<0.001 in comparison with control (WT).

We next addressed the role of C3a on pulmonary helper T cell responses upon allergenic challenge. To this end, we used C3aR-deficient (C3aR^−/−^) mice [62] (Figure 2A). We intranasally challenged C3aR^−/−^ and wild-type (C57BL/6) mice with Asp/OVA every other days (total 4 times). Twenty-four hours after the last challenge, the lungs, lung draining lymph nodes (dLNs), and BAL fluid were obtained and analyzed. When CD4^+^ T cells from the dLNs and the lungs were analyzed, we observed a small but evident population of IL-17-producing CD4^+^ T (Th17) cells (Figure 2B). Frequencies and absolute numbers of the IL-17-producing CD4^+^ T cells were significantly higher in the lungs and dLNs of C3aR^−/−^ mice than those of wild-type mice (Figure 2B & C and Table 1). Compared with wild-type mice, challenged C3aR^−/−^ mice exhibited a less cellularity in the dLNs (WT vs C3aR^−/−^: 11.07±1.28 vs 3.73±0.58 (×10^6^), *p* = 0.0064). We observed that the frequencies of IL-4 and IL-5 producing CD4^+^ T (Th2) cells were slightly higher in the dLNs of C3aR^−/−^ mice than those of wild-type mice; however, the absolute numbers of the Th2 cells were significantly lower in the dLNs of C3aR^−/−^ mice than wild-type mice (Table 1), due to the less cellularity in the C3aR^−/−^ mice.

**Table 1:** Analysis of IL-17-, IFNγ- or IL-4/5-producing CD4^+^ T cells in the draining LNs after intranasal allergen challenge.

	Frequency (%)			Absolute cells number (×10^4^)		
	IL-17^+^	IFNγ^+^	IL-4/5^+^	IL-17^+^	IFNγ^+^	IL-4/5^+^
Wild-type	0.574±0.12	0.358±0.07	0.411±0.06	6.301±0.72	3.955±0.46	4.551±0.52
C3aR^−/−^	5.988±0.77^**^	0.665±0.06^*^	0.735±0.19^*^	22.35±3.48^**^	2.47±0.39	2.71±0.39^*^

Groups of C3aR^−/−^ and wild-type mice (n = 4–5 per group) were intranasally administered with allergen every other days for four times. One day after the last challenge, lymphoid cells from the lung or draining LNs were obtained, and the expression of IL-17, IFN-γ, and IL-4/5 by CD4+ T cells was analyzed by intracellular staining. Data shown are mean ± SE after gated on CD4 expression, and represent two independent experiments. *, p<0.05 or **, p<0.01 in comparison with wild-type.

**Figure 3:**
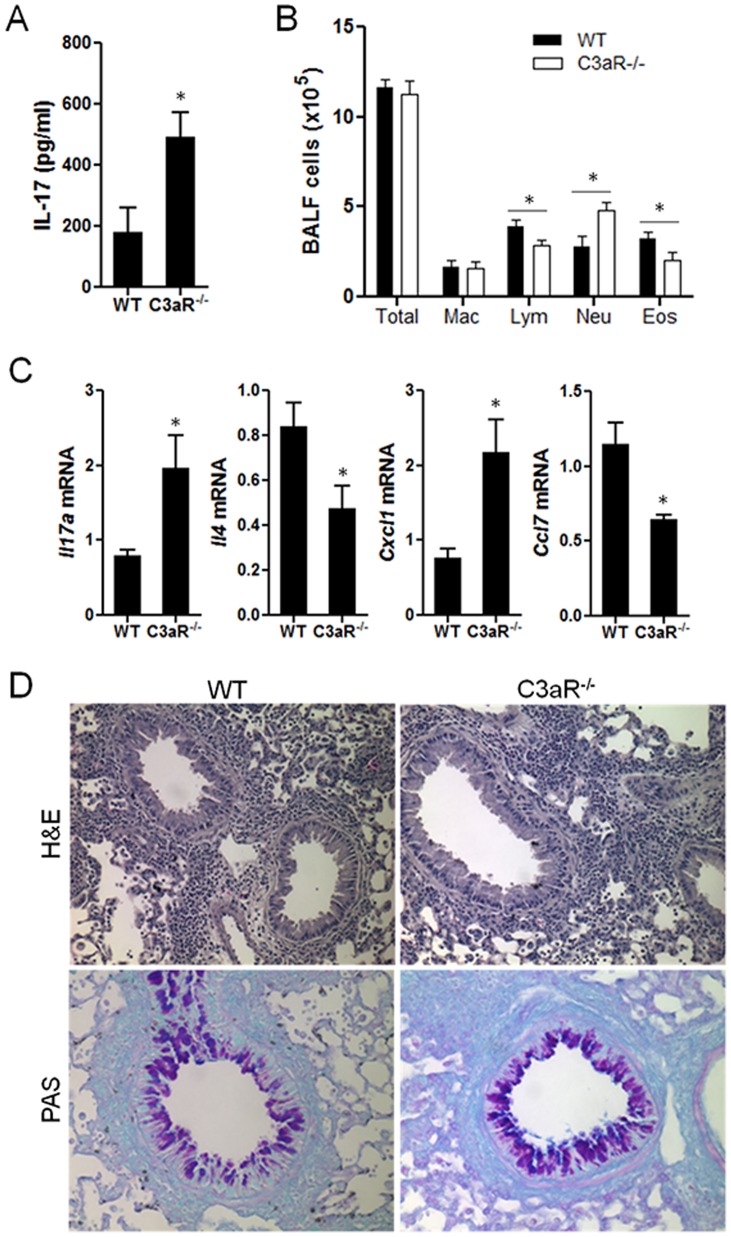
Increased IL-17 and neutrophils in the BAL fluid of C3aR-deficient mice after allergenic challenges. Groups of C3aR^−/−^ and wild-type mice (n = 4) were intranasally administered with allergen every other days for four times. One day after the last challenge, the lungs and BAL fluid were obtained. The concentration of IL-17 (*A*) and the numbers of macrophages, lymphocytes, eosinophils and neutrophils were evaluated (*B*). The levels of mRNA transcript of the indicated genes were determined by quantitative RT-PCR and were normalized with expression levels of *Actb* (*C*). Histology of the lungs were examined by H&E and periodic acid-Schiff (PAS) staining (×20 magnification) and visualized by light microscope (*D*). Data shown are mean ± SE, and represent two independent experiments. *, p<0.05 in comparison with WT control.

Consistent with the increased Th17 population in the lung and dLNs of C3aR^−/−^ mice, we observed a significantly higher concentration of IL-17 in the BAL fluid from C3aR^−/−^ mice than wild-type mice (Figure 3 A). To further determine the role of the increased Th17 population and IL-17 production in the lung of C3aR^−/−^ mice, we analyzed the cellular infiltration into the airway. The total cell number in the BAL fluid from C3aR^−/−^ mice was comparable to that of wild-type mice. Consistent with a previous study [52], the numbers of eosinophils and lymphocytes were lower in the C3aR^−/−^ mice than in the wild-type mice (Figure 3 B). On the contrary, we observed a significant increase in the number of neutrophils in the BAL fluid from C3aR^−/−^ mice compared with that of wild-type mice (Figure 3 B), which is well correlated with the increased Th17 population and IL-17 production in the airway of C3aR^−/−^ mice (Figure 2 and Table 1). CXCL1 is a potent chemoattractant for neutrophils and a well-known downstream of IL-17 signaling. Quantitative real-time PCR analysis showed that the increased expression of *Il17a* in the lung of C3aR^−/−^ mice is tightly associated with increased *Cxcl1* transcript, while the levels of *Il4* and *Ccl7* transcripts were significantly lower in the same mice (Figure 3C). To further characterize lung inflammation, we analyzed lung histology by hematoxylin and eosin (H&E) and periodic acid-Schiff (PAS) staining, and found similar infiltration of inflammatory cells and mucus-producing cells (Figure 3D) in our experimental setting. Taken together, these results demonstrate that C3aR^−/−^ mice exhibited an increased pulmonary Th17 response upon allergen challenge, associated with increased neutrophil infiltration into the airway.

### Allergen-specific Th17 responses in C3aR-deficient mice

**Figure 4:**
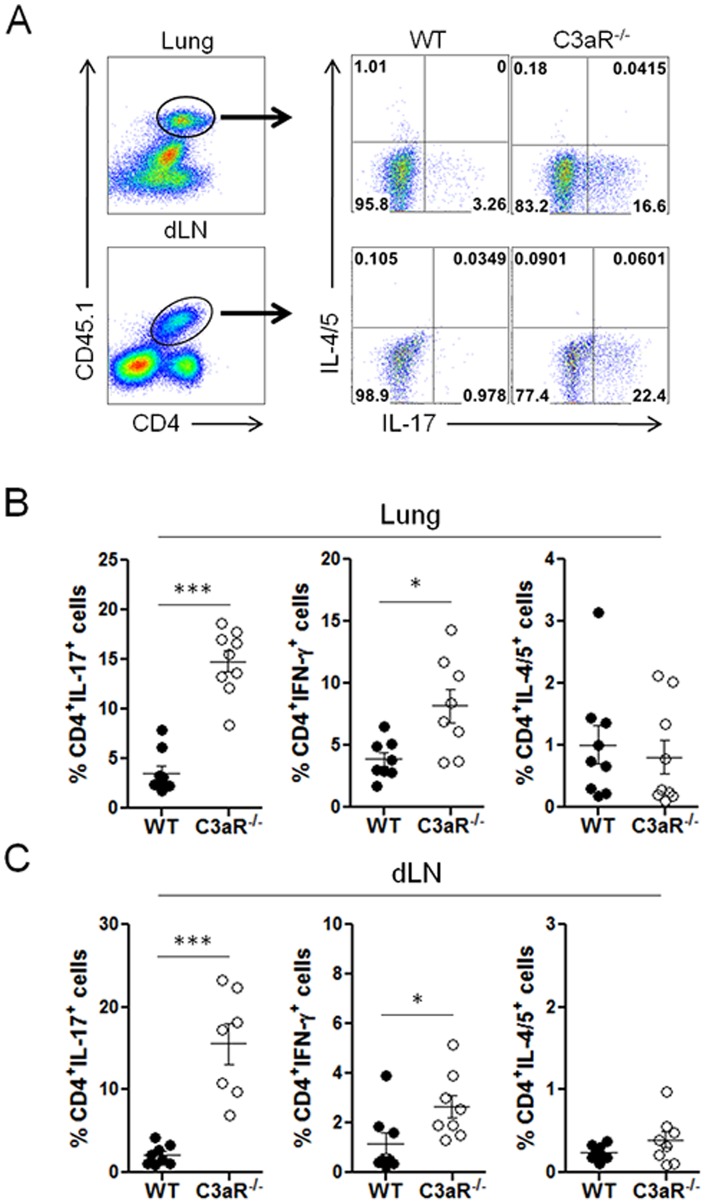
Antigen-specific pulmonary Th17 responses in C3aR-deficient mice. *A–C*, Groups of C3aR^−/−^ and wild-type mice (n = 7–9) were i.v. injected with CD45.1^+^ OT-II T cells (5×10^6^ cells/transfer; day -1), and were intranasally injected with the mixture of *Aspergillus* proteinase plus OVA on day 0, 2, 4, 6. On day 7, lymphoid cells from the lung (*B*) and draining LNs (*C*) were restimulated with PMA and ionomycin in the presence of brefeldin A and monensin, stained with anti-CD45.1 and CD4, and the expression of IL-17, IFN-γ, or IL-4 + IL-5 by CD45.1^+^CD4^+^ donor T cells was analyzed by intracellular staining. Left panels in *A* illustrate gating strategy. Bars in *B* and *C* show the mean values. Data shown represent at least three independent experiments. *, p<0.05 or ***, p<0.001 in comparison with wild-type recipients.

Although we observed an increased Th17 population in the lungs of the challenged C3aR^−/−^ mice, it was not clear if C3a signaling affects the generation of ‘allergen-specific’ Th17 responses. To address this point, we employed ovalbumin-specific TcR-transgenic (OT-II) T cells that express CD45.1 as a congenic marker. We adoptively transferred CD45.1^+^ OT-II T cells into C3aR^−/−^ and wild-type mice (CD45.2^+^), and the recipients were intranasally challenged with Asp/OVA. This system allowed us to specifically track allergen-specific CD4^+^ T cell responses by analyzing the CD45.1^+^ donor cells (Figure 4 A, *left* panels). As shown in Figure 4 A & B and Figure S1, we observed a significant increase in the IL-17^+^ population among the donor T cells in the lungs of the C3aR^−/−^ mice compared to the wild-type mice (WT vs C3aR^−/−^: 3.544±0.686 vs 14.77±1.076, *p*<0.0001). We also observed a slight increase in IFN-γ^+^ Th1 cell population in the C3aR^−/−^ mice (WT vs C3aR^−/−^: 3.86±0.557 vs 8.166±1.352, *p* = 0.0148), while Th2 cell number appeared to be comparable (WT vs C3aR^−/−^: 1.007±0.308 vs 0.809±0.272, *p* = 0.4894). Moreover, the increased allergen-specific Th17 cell population was also evident in the dLNs of the C3aR^−/−^ mice (WT vs C3aR^−/−^: 2.161 0.425 vs 15.53 2.411, *p*<0.0001) (Figure 4 C).

As depicted in Figure 4B, Th1 and Th17 populations from the allergen-specific donor cells were comparable to the wild-type recipients (Th1 vs Th17: 3.86±0.557 vs 3.544±0.686, *p* = 0.7303). However, in the C3aR^−/−^ recipients, the frequency of Th17 cells was significantly higher than that of Th1 cells (Th1 vs Th17: 8.166±1.352 vs 14.77±1.076, *p* = 0.0015). This difference in the Th1-Th17 ratio was even more dramatic in the dLNs (Th1 vs Th17: 2.656±0.467 vs 15.53±2.411, *p*<0.0001). In addition, we also observed a significantly increased Th17 cell population among CD45.2^+^CD4^+^ host T cells in the lungs of the C3aR^−/−^ mice compared with wild-type mice (Figure S2). These results collectively demonstrate that signaling through the C3aR suppressed the generation of antigen-specific Th17 cells in the lung and draining lymph nodes after allergen challenge.

### C3aR signal on hematopoietic cells inhibit pulmonary Th17 responses

C3aR is expressed on certain parenchymal cells, including epithelial and endothelial cells [43], [44], as well as on hematopoietic cells, including macrophages, dendritic cells, and eosinophils [45], [46], [47], [48]. During lung inflammation, C3a/C3aR mediated signaling in lung epithelial cells induces the expression of Muc5ac [67]. In addition, C3a induces the expression of IL-8 from an epithelial cell line [68]. Reports have also shown that C3a regulates helper T cell responses by modulating the function of antigen presenting cells [5], [69]. To further characterize the mechanism of C3a-mediated suppression of pulmonary Th17 responses, we sought to determine whether C3aR expression on hematopoietic or parenchymal cells is required to suppress the allergen-specific Th17 responses in the lung. To this end, we generated bone marrow reconstituted mice by transferring bone marrow cells from wild-type or C3aR^−/−^ mice into lethally irradiated wild-type or C3aR^−/−^ recipients. This system allowed us to establish *in vivo* models where C3aR is solely expressed either on hematopoietic cells (BM^WT^→KO) or on parenchymal cells (BM^KO^→WT). Six to eight weeks after the reconstitution, the recipient mice were additionally injected with CD4^+^ T cells from CD45.1^+^ OT-II mice, followed by intranasal challenges with Asp/OVA.

**Figure 5:**
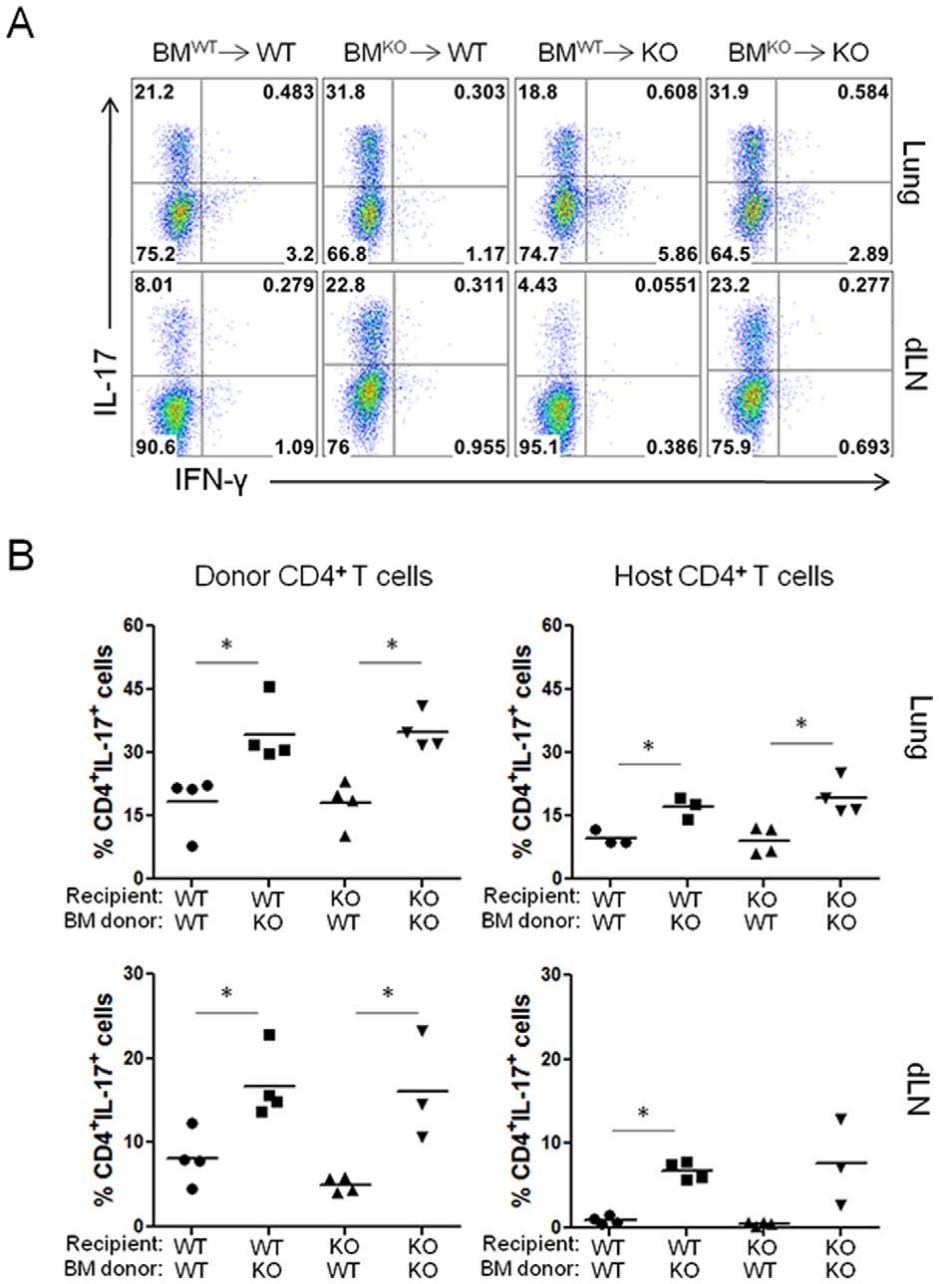
C3aR-deficiency on hematopoietic cells induces the increased pulmonary Th17 responses. Bone marrow cells from wild-type or C3aR^−/−^ mice were adoptively transferred into wild-type or C3aR^−/−^ mice (n = 3–4 per group; 5–10×10^6^ cells/transfer). Six to eight weeks later, the reconstituted mice were injected i.v. with CD45.1^+^OT-II T cells, and were further challenged with intranasal allergen every other day for a total of four times. One day after the final challenge, the expression of IL-17 and IFN-γ by CD45.1^+^ donor CD4^+^ T cells in the lungs and draining lymph nodes was analyzed by flow cytometry. Bars in *B* are mean values. Data shown represent two independent experiments. *, p<0.05 in comparison with WT bone marrow reconstituted mice.

As shown in Figure 5, we observed that the percentage of Th17 cells in the lung among the CD45.1^+^ population in wild-type mice reconstituted with C3aR^−/−^ bone marrow (BM^KO^→WT) was comparable to that of C3aR^−/−^ mice receiving C3aR^−/−^ bone marrow (BM^KO^→KO) but was significantly higher than that of wild-type mice receiving wild-type bone marrow (BM^WT^→WT) (Figure 5A & B). On the other hand, the Th17 population in the ‘BM^WT^→KO’ mice was significantly lower than that of both ‘BM^KO^→WT’ and ‘BM^KO^→KO’ mice. We observed very similar results in the CD45.1^+^ population from draining LNs (Figure 5A & B). When we analyzed the endogenous CD4^+^ T cells (CD45.1^−^), we observed higher frequencies of IL-17^+^ cells in the recipients of C3aR^−/−^ bone marrow regardless of the recipient background (data not shown). We repeatedly observed that the percentages of IL-17^+^ CD4^+^ T cells in the lung were higher in the bone-marrow reconstituted mice compared with those in non-irradiated mice as shown in Figure 4. Collectively, these data strongly suggest that C3aR expression on hematopoietic cells is critical for the C3a-mediated suppression of pulmonary Th17 responses against allergens in this animal model.

### Foxp3^+^ regulatory T cells are increased in C3aR^−/−^ mice but not required for pulmonary Th17 responses

**Figure 6:**
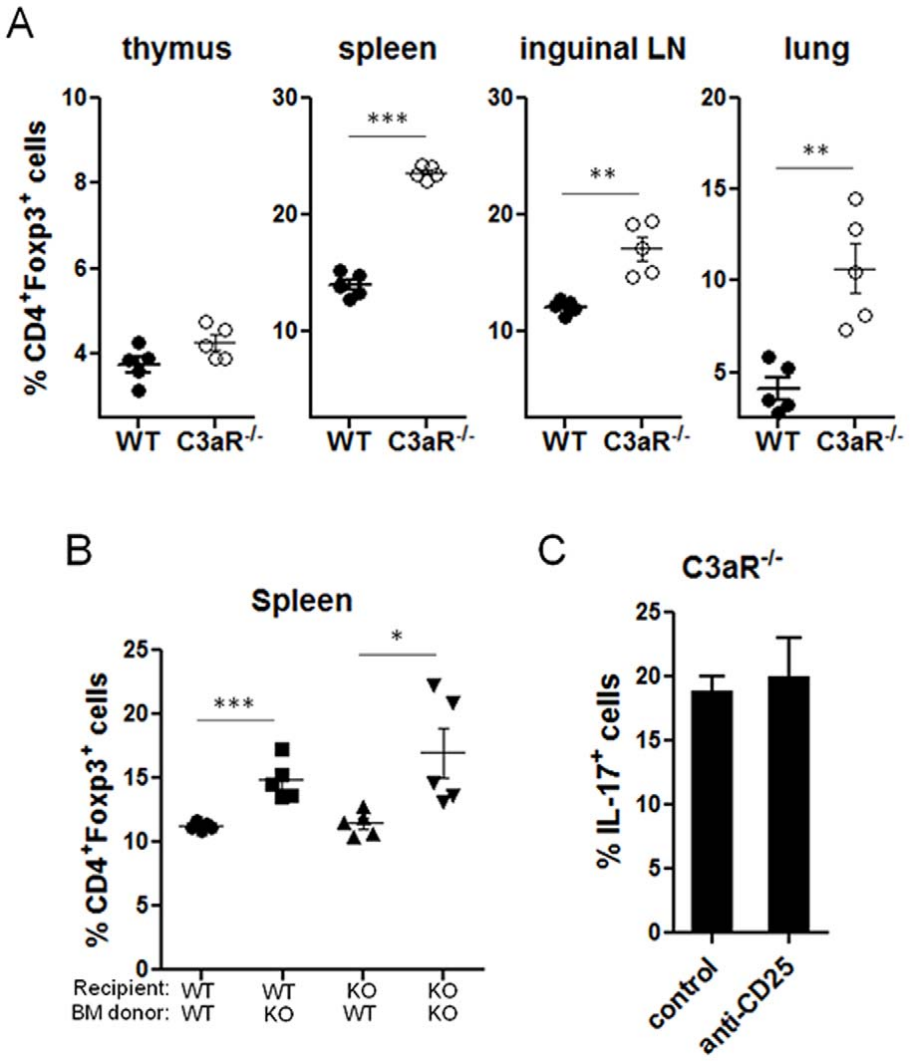
Foxp3^+^ regulatory T cells in C3aR^−/−^ mice. *A* and *B*, Lymphoid cells from the indicated organs were obtained from wild-type and C3aR^−/−^ mice (*A*), or the spleens from bone-marrow reconstituted mice (*B*) generated as described in *Figure 5*. The percentage of Foxp3^+^ cells among CD4^+^ (spleen, lung, inguinal lymph node (iLN)) or CD4^+^CD8^−^ (thymus) cells was analyzed by flow cytometry. *C*, Groups of C3aR^−/−^ mice were intraperitoneally injected with control IgG or anti-CD25 (PC-61) (n = 3–4) on day −2, day 0, and day 2. The mice were intravenously injected with CD45.1^+^ OT-II T cells on day -1, followed by subsequent intranasal injection with Asp/OVA on day 0, 2, 4, and 6. On day 7, lymphoid cells from the lung or draining lymph nodes were obtained, and the expression of IL-17 by CD4^+^ T cells was analyzed by intracellular staining. Data shown represent three (*A*) or two (*B* & *C*) independent experiments. *, p<0.05 or **, p<0.01 in comparison with WT (*A*), or isotype control IgG treatment (*C*).

Two recent studies showed an important role of Foxp3^+^ regulatory T (Treg) cells on Th17 differentiation through consumption of IL-2 [70] or by providing TGF-β [71]. The possibility that C3a signals impact Treg cells response has not been examined. During the course of our analysis, we found that the percentages of CD4^+^Foxp3^+^ regulatory T cells in C3aR^−/−^ mice was significantly higher than those of wild-type mice in all secondary lymphoid organs and the lung but not in the thymus (Fig. 6 A), indicating that C3a signaling regulates the size of Treg cells in the periphery or the induction of induced Treg cells. This inhibition of peripheral Treg population by C3a appeared to be dependent on the expression of C3aR on hematopoietic cells, since wild-type mice reconstituted with C3aR^−/−^ bone marrow also showed an increased percentage of Treg cells in the spleens (Figure 6 B).

To test whether the increased Treg population accounted for the observed increased pulmonary Th17 responses in the C3aR^−/−^ mice, we injected anti-CD25 Ab that has been known to deplete the majority of Treg cells [63], or control Ab into C3aR^−/−^ mice before adoptive transfer of CD45.1^+^OT-II T cells and subsequent intranasal challenges with Asp/OVA. As shown in Figure 6 C, we observed little difference in the frequency of Th17 cells between control Ab- and anti-CD25-treated C3aR^−/−^ mice.

To evaluate the mechanisms of how a lack of C3aR expression mediates enhancement of IL-17-producing CD4^+^ T cells in the lung after allergen challenge, we determined the levels of cytokines that are known to promote Th17 cell differentiation and maintenance [9], [10], [11], [12]. *Il23a* (encoding IL-23p19) expression was significantly increased in the lungs of challenged C3aR^−/−^ mice compared to challenged wild-type mice, while the expression of *Il12a* (encoding IL-12p35), *Il12b* (encoding IL-12/IL-23p40), *Il1b*, *Il6*, *Il10*, and *Csf2* (encoding GM-CSF) in the lungs were not significantly different between C3aR^−/−^ and wild-type mice (Figure S3A). Compared to wild-type mice, the concentration of TGF-β and IL-23 were slightly but significantly increased in C3aR^−/−^ mouse lung homogenates after allergen challenge (Figure S3B), while IL-6 protein level was not significantly different. To test the possible involvement of IL-23 in the enhanced pulmonary Th17 responses in the C3aR^−/−^ mice, we intraperitoneally injected anti-p19 neutralizing antibody or control IgG into the C3aR^−/−^ mice during intranasal allergenic challenges, and found no significant difference in the frequency of the Th17 population in the lungs and dLNs between the two treatments (data not shown). Overall, these data demonstrate that, despite a higher Treg population and increased IL-23, the increased pulmonary Th17 responses in the C3aR^−/−^ mice were likely through a Treg- and IL-23-independent manner.

### Neutralization of IL-17 reduces the infiltration of neutrophils into airway in C3aR-deficient mice

**Figure 7:**
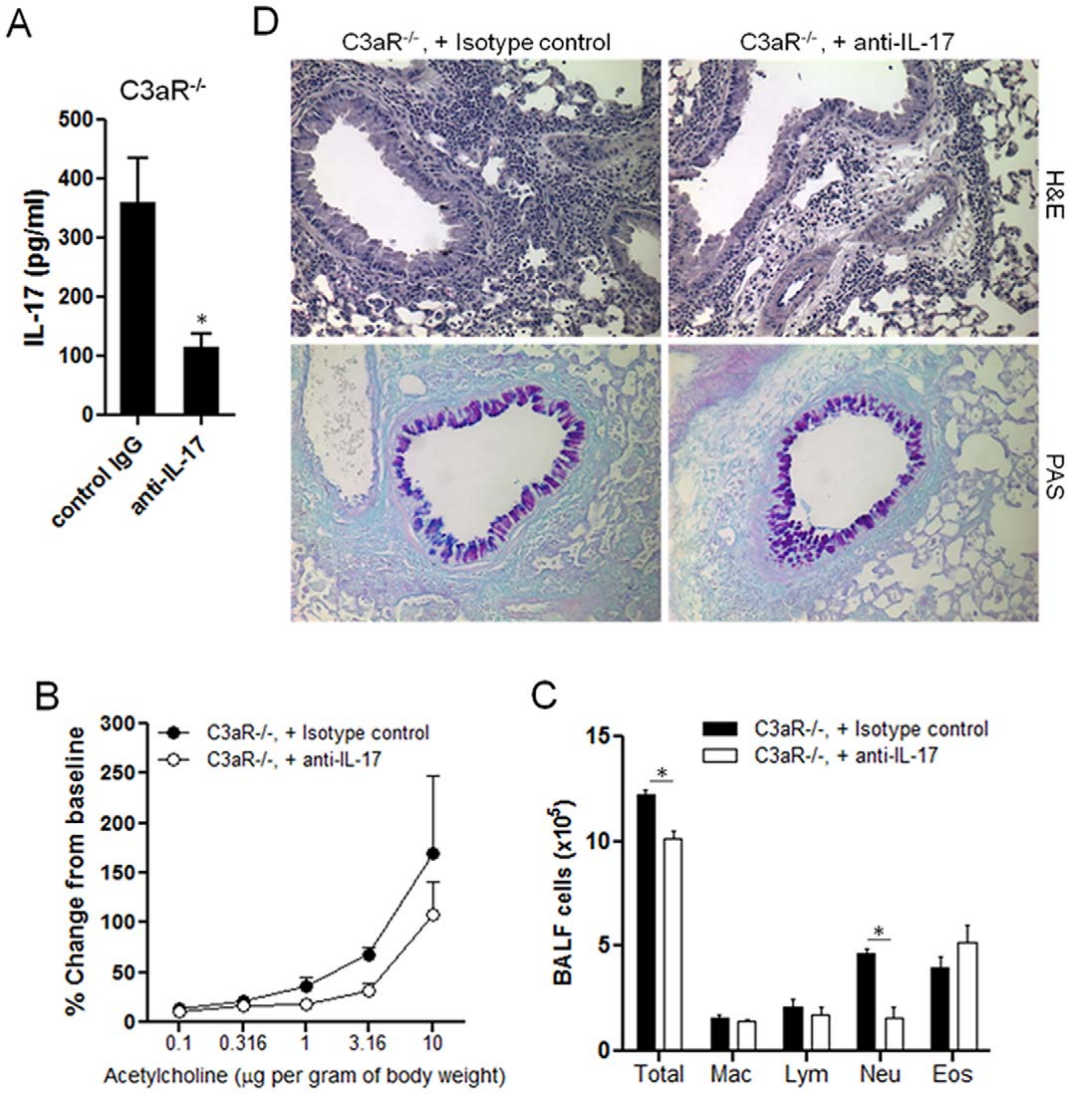
Effect of IL-17 neutralization on the lung inflammation of C3aR^−/−^ mice. Groups of C3aR^−/−^ mice were (n = 3–4) intranasally challenge with Asp/OVA every other days four times (day 0, 2, 4, 6), and i.p. injected with isotype control antibody or with IL-17 neutralizing antibody on day 0, 2, 4. Twenty-four hours after the last challenge, mice were anesthetized, mechanically ventilated, and airway responses to increasing doses of intravenous acetylcholine were measured. AHR is expressed as the percentage of changes from baseline (*B*). The BAL fluid and lungs were collected, and the amounts of IL-17 (*A*) and number of the indicated cell population (*C*) in the BAL fluid were measured. Histology of the lungs was examined by H&E and PAS staining (×20 magnification) and visualized by light microscope (*D*). Data shown are mean ± SE. *, p<0.05 in comparison with control antibody treated C3aR^−/−^ mice.

To directly determine the role of increased IL-17/Th17 responses in the lung of C3aR^−/−^ mice, we intraperitoneally injected anti-IL-17 neutralizing antibody or control IgG into the C3aR^−/−^ mice during intranasal challenges. Twenty-four hours after the last challenge, the mice were subjected to measure airway hyperresponsiveness (AHR) to increasing doses of intravenous acetylcholine (ACh) [52]. As expected, anti-IL-17 treatment decreased the amounts of IL-17 in BAL fluid (Figure 7A). C3aR^−/−^ mice have been reported to be resistant to allergen-induced AHR [52]. Although the C3aR^−/−^ mice treated with anti-IL-17 showed slightly lower AHR compared with isotype-treated mice, it did not reach statistical significance (Figure 7B), suggesting little role of IL-17 in regulating airway reactivity in C3aR^−/−^ mice. Notably, however, anti-IL-17 treatment significantly decreased the total number of cells in BAL fluid, mainly due to the significant decreased number of neutrophils (Figure 7C). Histologic examination of the lungs also showed a mild decrease of inflammatory cell infiltration in mice treated with anti-IL-17 (Figure 7D, upper panels). We observed comparable PAS staining between anti-IL-17- and isotype-treated C3aR^−/−^ mice, indicating little role of IL-17 in mucus production. These results together demonstrate that the increased IL-17/Th17 responses in the C3aR^−/−^ directly contributed to the neutrophil infiltration into airway, and thus suggest that C3a suppresses neutrophilic lung inflammation against the *Aspergillus* proteinase allergen by inhibiting pulmonary Th17 responses *in vivo*.

## Discussion

In the present study, we examined the role of C3a on shaping pulmonary helper T cell responses in an animal model of allergic lung inflammation. We observed that C3aR-deficient mice exhibited a higher frequency of Th17 cells in the lung compared to wild-type mice upon intranasal allergenic challenges. Similarly, adoptive transfer studies showed that C3aR-deficiency facilitated the differentiation of antigen-specific naïve CD4^+^ T cells into Th17 cells in the lung and draining lymph nodes. Bone-marrow transfer studies revealed that C3aR-deficiency on hematopoietic cells was required for the increased pulmonary Th17 responses in the C3aR-deficient mice. Although C3aR-deficient mice had an increased Foxp3^+^ Treg population, depletion of Treg cells in the C3aR-deficient mice minimally affected the generation of pulmonary Th17 cells. Histologic examination and BAL fluid analysis showed that C3aR-deficient mice exhibited increased neutrophils in the airway, and that anti-IL-17 treatment specifically reduced the number of neutrophils in BAL fluid with little effects on airway reactivity and mucus production. Our results thus demonstrate that C3aR signaling on hematopoietic cells suppresses the generation of allergen-specific Th17 cells in the lung and neutrophilia in the airway via a Treg independent mechanism.

Contribution of Th2 cells and their cytokines to allergic asthma has been well described [1], [2]. In addition, growing evidence has demonstrated the non-redundant role of Th17 cells and their cytokines in allergic lung inflammation. In particular, Th17 cells have been proposed to mediate neutrophilic lung inflammation [4], [6] as well as steroid resistant severe form of asthma [32]. Moreover, the clinical severity of asthma is tightly associated with the amount of IL-17 in sputum and circulation in humans [23], [24], [25]. Therefore it is likely that Th2 and Th17 responses mediate different forms of allergic lung inflammation (eosinophilic vs neutrophilic), or are important regulators at different stages during lung inflammation. Although fungal-associated allergic proteinases has been shown to induce neutrophil recruitment into the airway independently of T cells and C3 signal [72], the increased Th17 population and elevated neutrophils in C3aR^−/−^ mice in the present study strongly suggest that C3a signal inhibits allergen-induced neutrophilia in the airway, probably via inhibition of pulmonary Th17 responses. Our study provides experimental evidence that blockade of C3aR signal may induce increased Th17 responses and neutrophilia in the airway. A recent study has attempted to use C3aR antagonist for the treatment of allergic lung inflammation in mice [73]. However, based on the present study, the use of C3aR antagonist may induce neutrophilic lung inflammation, and thus more cautious consideration will be needed for the use of C3a/C3aR antagonist in clinical setting. A recent study by Wang *et al* has elegantly shown the existence of Th2/Th17 cells expressing both GATA3 and RORγt in inflamed lung [74]. However, the IL-17-producing CD4^+^ T cells in the lung of C3aR^−/−^ mice in the present study did not co-express IL-4 or IL-5, indicating that they are Th17 rather than Th2/Th17 cells (Figure 4 A).

In addition to its well-known functions in host defense, the role of the complement system in adaptive immunity is only in the past several years been fully appreciated. For instance, complement C5a has been shown to promote Th17-mediated autoimmune arthritis in SKG mice and experimental autoimmune encephalomyelitis by inducing IL-6 from antigen-presenting cells [75], [76], [77]. On the other hand, it has been shown that C5aR-deficient DCs produce higher amounts of TGF-β and facilitate the generation of Th17 cells, indicating an inhibitory role of C5a on Th17 responses [78]. More recently, C5a has also been reported to suppress the production IL-17 and IL-23 from macrophages in an animal model of septic shock by inducing IL-10 [79]. In addition, Lajoie *et al* have recently described the opposing role of C3a and C5a in regulating Th17 responses in an animal model of allergic asthma induced by house dust mite extract [5]. They showed that C3a stimulates IL-23 production from dendritic cells, and that C3aR-deficient mice had fewer Th17 cells in the airway in their asthma model. By sharp contrast, we observed an increased Th17 population in the lungs of C3aR-deficient mice upon intranasal challenge with *Aspergillus* allergen. By using CD45.1^+^OT-II CD4^+^ T cells adoptive transfer studies, we convincingly showed that C3aR signal suppresses the generation of ‘antigen-specific’ Th17 cells in the lung. Although it is unclear how C3a exerts these contradictory functions at this stage, it is possible that the function of C3a depends on or the duration of inflammation (acute vs. chronic). In this aspect, it is notable that prostaglandin E2 can suppress IL-17 production during early Th17 differentiation, but enhance IL-17 production by mature Th17 cells [80]. Since C3a have been shown to induce the production of prostaglandin E2 by macrophage [81], [82], it is feasible to surmise that C3a-prostaglandin E2 pathway differently affects Th17 cells depending on their differentiation stages. Further studies are needed to clearly define the role of C3a in regulating pulmonary Th17 responses. It is also possible that the discrepancy between the two studies is due to different nature of allergen used. We used the mixture of *Aspergillus* proteinase and OVA as our model allergen whereas the prior study by Lajoie *et al* used house dust mite extract containing containing about 20 % weight of protein including Der p1 as well as non-protein components such as endotoxin [5]. Therefore, we speculate that the mechanism of C3a induction by these two allergens might be different. For instance, while proteinase activity seems crucial for C3a production by allergen challenge in the present study (Figure 1D), it is less clear how house dust mite extract induced C3a *in vivo*. Another possible explanation is that, in addition to C3a, these two allergens might induce different innate cytokines which overall could lead to different outcome in helper T cell responses *in vivo*.

Our bone-marrow reconstitution study clearly demonstrates that C3aR expression on hematopoietic cells is responsible for the increased pulmonary Th17 responses. It is not likely that the C3a signal on CD4^+^ T cells suppresses Th17 polarization, since we also observed the increased Th17 population among the wild-type donor T cell population as well as the host CD4^+^ T cells in the C3aR^−/−^ recipients (Figure 4). Several recent studies demonstrated that C3a and C5a indirectly affect helper T cell responses by modulating the function of antigen-presenting cells [5], [69], [77], [78]. When we compared dendritic cells (DCs) obtained from wild-type and C3aR-deficient mice in a T cell-DC co-culture system in the presence of allergen or LPS plus TGF-β [12], [83], we observed that C3aR-deficient DCs induced a lower percentage of Th17 cells than wild-type DCs (data not shown), suggesting that this *in vitro* T cell-DC co-culture system did not account for the *in vivo* phenotype of C3aR-deficient mice. The type(s) of C3aR-expressing immune cells responsible for the increased Th17 responses in C3aR^−/−^ mice is not clear at this stage. It is possible that C3a suppresses Th17 responses by stimulating innate immune cells other than antigen-presenting cells, such as neutrophils, mast cells, or eosinophils as they also express C3aR [43], [44], [45], [46], [47], [48].

Another novel finding in this study is that the C3aR-deficient mice had an increased percentage of Foxp3^+^ Treg cells in the secondary lymphoid organs, but not in the thymus, suggesting a crucial function of C3aR signaling in regulating the size of the Treg population in the periphery. Since Treg cells have been reported to promote Th17 differentiation [70], [71], we tested if the increased Treg cells could account for the enhanced pulmonary Th17 responses in the C3aR-deficient mice but found little effect of Treg depletion on the generation of Th17 cells in the lung. Therefore, C3a inhibited pulmonary Th17 responses in a Treg independent manner in our experimental setting. We also observed higher amounts of TGF-β in the lung of C3aR^−/−^; however, the exact role of the increased TGF-β in pulmonary Th17 and Treg is not clear at this stage. In summary, our study demonstrates that C3aR signaling on hematopoietic cells negatively regulates the generation of allergen-specific Th17 cells in the lung, and that it down-regulates the size of the Foxp3^+^ Treg population in the periphery. These findings unveil a critical role of C3a in balancing adaptive T cell responses during pulmonary inflammation *in vivo*.

## Supporting Information

Figure S1 **Allergen-specific Th1, Th2, and Th17 responses in the lung after allergenic challenge.** C3aR^−/−^ mice were i.v. injected with CD45.1^+^OT-II T cells (day −1), and were intranasally injected with Asp/OVA allergen on day 0, 2, 4, 6. On day 7, lymphoid cells obtained from the lung, and the expression of IL-17A, IFN-γ or IL-4/5 by donor CD4^+^ T cells was analyzed by intracellular staining. Data shown are gated on CD45.1^+^ CD4^+^ cells.
(TIF)

Figure S2 **Host CD4^+^ T cell responses in C3aR-deficient mice.** Groups of C3aR^−/−^ and wild-type mice (n = 7–9) were i.v. injected with CD45.1^+^ OT-II T cells (day −1), and were intranasally injected with *Aspergillus* allergen plus OVA (Asp/OVA) on day 0, 2, 4, 6. On day 7, lymphoid cells from the lung (*A*) and draining LNs (*B*) were stained with anti-CD45.1 and CD4, and the expression of IL-17, IFNγ, or IL-4 + IL-5 by CD45.1^−^CD4^+^ host T cells was analyzed by intracellular staining. Bars in *A* and *B* show the mean ± SE values. *, p<0.05 or ***, p<0.001 in comparison with wild-type recipients.
(TIF)

Figure S3 **Expression of cytokines in the lung of C3aR-deficient mice upon allergenic challenge.** C3aR^−/−^ and wild-type mice were intranasal Injected with *Aspergillus* proteinase allergen one time, and obtained lung cells and homogenate after 24 hours. The mRNA expression of indicated genes were analyzed by quantitative RT-PCR (*A*) and protein levels in lung homogenates were evaluated by ELISA (*B*). Data shown represent at least two independent experiments. *, p<0.05 or **, p<0.01 in comparison with wild-type mice.
(TIF)
