# Supplementary figures and images for: Negative Regulation of Pulmonary Th17 Responses by C3a Anaphylatoxin during Allergic Inflammation in Mice
Source: PLoS One. 2012 Dec 20;7(12):e52666. doi: 10.1371/journal.pone.0052666 (PMC3527591; doi:10.1371/journal.pone.0052666)

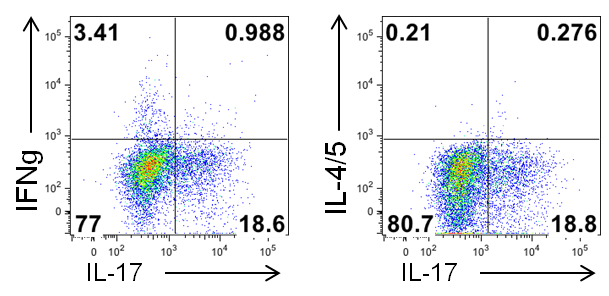

Supplement: Figure S1 — Allergen-specific Th1, Th2, and Th17 responses in the lung after allergenic challenge. C3aR−/− mice were i.v. injected with CD45.1+OT-II T cells (day −1), and were intranasally injected with Asp/OVA allergen on day 0, 2, 4, 6. On day 7, lymphoid cells obtained from the lung, and the expression of IL-17A, IFN-γ or IL-4/5 by donor CD4+ T cells was analyzed by intracellular staining. Data shown are gated on CD45.1+ CD4+ cells. (TIF) [file pone.0052666.s001.tif]

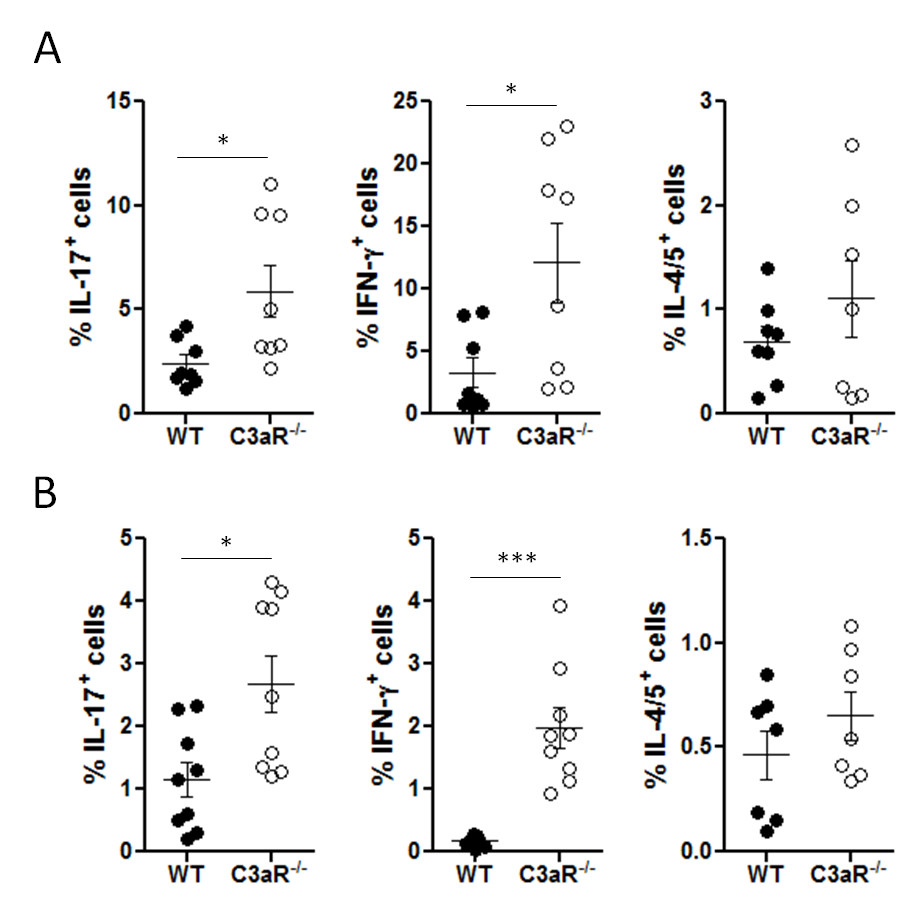

Supplement: Figure S2 — Host CD4+ T cell responses in C3aR-deficient mice. Groups of C3aR−/− and wild-type mice (n = 7–9) were i.v. injected with CD45.1+ OT-II T cells (day −1), and were intranasally injected with Aspergillus allergen plus OVA (Asp/OVA) on day 0, 2, 4, 6. On day 7, lymphoid cells from the lung (A) and draining LNs (B) were stained with anti-CD45.1 and CD4, and the expression of IL-17, IFNγ, or IL-4 + IL-5 by CD45.1−CD4+ host T cells was analyzed by intracellular staining. Bars in A and B show the mean ± SE values. *, p<0.05 or ***, p<0.001 in comparison with wild-type recipients. (TIF) [file pone.0052666.s002.tif]

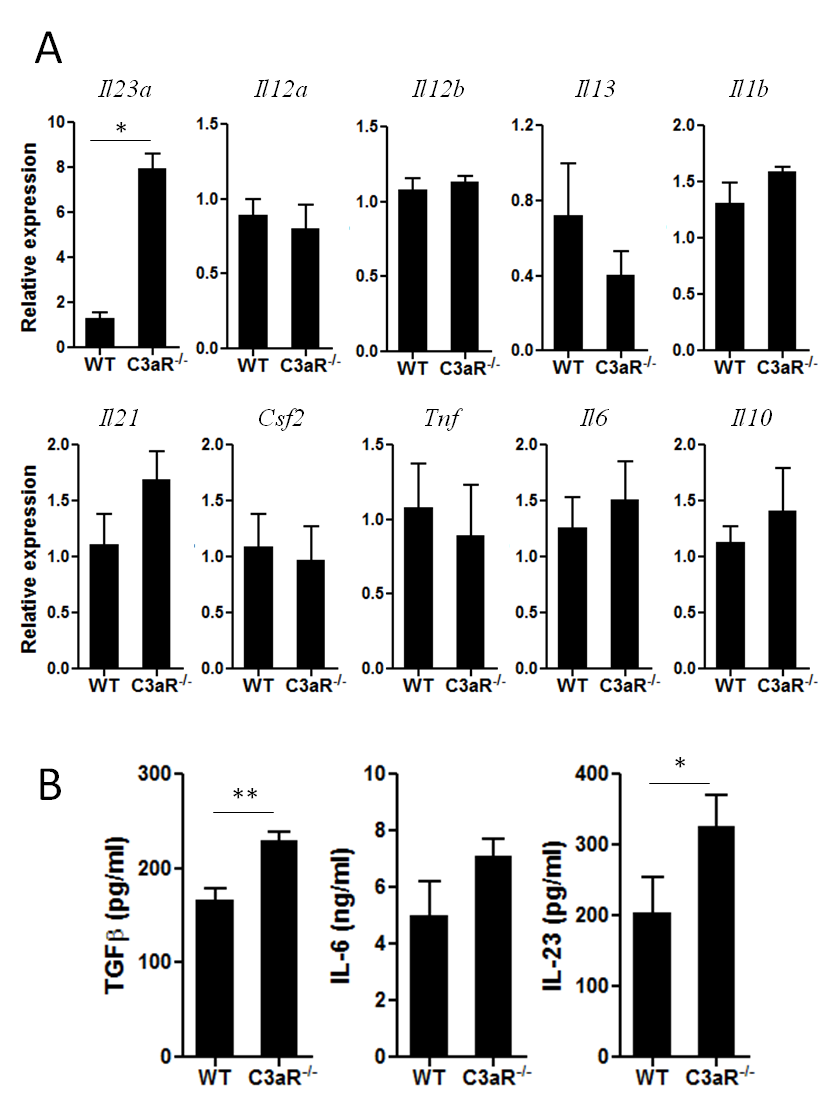

Supplement: Figure S3 — Expression of cytokines in the lung of C3aR-deficient mice upon allergenic challenge. C3aR−/− and wild-type mice were intranasal Injected with Aspergillus proteinase allergen one time, and obtained lung cells and homogenate after 24 hours. The mRNA expression of indicated genes were analyzed by quantitative RT-PCR (A) and protein levels in lung homogenates were evaluated by ELISA (B). Data shown represent at least two independent experiments. *, p<0.05 or **, p<0.01 in comparison with wild-type mice. (TIF) [file pone.0052666.s003.tif]
